# Induced Fetal Demise Prior to Later Medication Abortion: A Qualitative Study of Women's Experience With Digoxin Injection

**DOI:** 10.1002/puh2.70087

**Published:** 2025-07-29

**Authors:** Abraham Fessehaye Sium, Abrham Getachew, Tesfaye Tufa

**Affiliations:** ^1^ Department of Obstetrics and Gynecology St. Paul's Hospital Millennium Medical College Addis Ababa Ethiopia; ^2^ Research Directorate Office St. Paul's Hospital Millennium Medical College Addis Ababa Ethiopia

**Keywords:** digoxin, digoxin injection, digoxin patient experience, feticide, induced fetal demise

## Abstract

**Background:**

Recent evidence suggests that providers who continue to use digoxin for induced fetal demise prior to later medication abortion should consider patient preferences in how they offer digoxin and consider tools to ensure patient understanding. This study aimed to qualitatively determine patients’ experiences with induced fetal demise using digoxin injection prior to later medication abortion.

**Methods:**

This was a qualitative study of women who had induced fetal demise with digoxin injection prior to later medication abortion in Ethiopia, in 2023. The participants were interviewed about physical and emotional experiences with digoxin and their understanding of its purpose in the immediate post‐abortion period, before they were discharged from the hospital. Data were analyzed using a thematic analysis on One Code software.

**Results:**

A total of 20 participants were included in this study. The two overarching themes identified from the qualitative interviews were as follows: (1) emotional discomfort and varied understanding of digoxin's purpose; (2) digoxin injection‐related physical discomfort. Participants had a shared feeling of discomfort carrying a demised fetus after digoxin administration till the abortion process was completed. A significant proportion of them (8/20, 40%) described induced fetal demise as a procedure to shorten the abortion process.

**Conclusions:**

Findings of some patients’ physical and emotional discomforts of digoxin injection to induce fetal demise in our study are consistent with findings in previous qualitative studies.

## Graphical abstract

We qualitatively determined patients’ experiences with induced fetal demise using digoxin injection prior to later medication abortion in Ethiopia. We identified two overarching themes: (1) emotional discomfort and varied understanding of digoxin's purpose; (2) digoxin injection‐related physical discomfort.

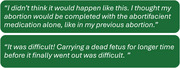

## 1 Introduction

The reasons for inducing fetal demise prior to later medication abortion are fears of legal retaliation; comfort of the patient, provider, and/or other involved health care workers; the belief that (D&E) will be easier and faster; to avoid transient fetal survival after medical induction; and to avoid extramural delivery with signs of life [1, 2, 3, 4, 5]. Among these reasons, avoidances of transient fetal survival after medication abortion and extramural delivery with signs of life are the most important reasons in the Ethiopian setting, where medication abortion is the most frequent method of later abortion [6]. The World Health Organization (WHO) abortion guidelines recommend consideration of fetal demise in medical termination of pregnancy above 20 weeks of gestation [7].

The efficacy of intra‐fetal and/or intra‐amniotic digoxin, intracardiac KCL, and intracardiac lidocaine in achieving pre‐abortion fetal demise has been supported with strong evidence. However, too few studies have evaluated patients’ preferences and acceptability of these different feticidal agents, for example, digoxin versus lidocaine [8]. A recent qualitative study suggested that providers who continue to use digoxin should consider patient preferences in how they offer digoxin and consider tools to ensure patient understanding [9]. The risks associated with digoxin have been well documented and include nausea and vomiting, increased risk of hospital admission in the period between injection and the scheduled abortion [10, 11].

At St. Paul's Hospital Millennium Medical College (SPHMMC) in Addis Ababa, Ethiopia, patients who present for later abortion are routinely provided with induced fetal demise before abortion. In our setting, resident physicians provide intraamniotic digoxin, the most commonly utilized option of induced fetal demise. This study sought to qualitatively understand patients’ experiences with induced fetal demise using digoxin injection as a step before later medication abortion.

## 2 Methods

### 2.1 Study Design, Period, and Setting

We conducted a qualitative study on women's experience of induced fetal demise with digoxin injection prior to later medication abortion procedures. It was conducted at SPHMMC, in Ethiopia, in March 2023. SPHMMC is a national referral center for complex family planning, including comprehensive abortion care, and it nests the only family planning fellowship training program in Africa. The college is among the leading tertiary teaching hospitals in Ethiopia.

In our center, SPHMMC, the available options of inducing fetal demise prior to later medication abortion are intra‐amniotic digoxin and intracardiac lidocaine. Although digoxin is the most commonly used option, decision on using either option of induced fetal demise is dependent on provider's preference and technical difficulty of administering digoxin in some cases. We aimed to qualitatively understand these patients’ experiences with digoxin as a step prior to later medication abortion. We defined later medication abortion as medical abortion care at gestational age of 20–28 weeks, in which cases inducing fetal demise is indicated in Ethiopia.

### 2.2 Data Collection

Patients who underwent later medication abortion cases with induced fetal demise with intra‐amniotic digoxin were interviewed on their experience with this induced fetal demise in the immediate post‐abortion period, before they were discharged from the hospital. The patients were consecutive cases that agreed to be interviewed during the first 3 weeks of March of 2023. We interviewed the patients about physical and emotional experiences with digoxin and understanding of its purpose.

We conducted 15–20 min, semi‐structured, qualitative face‐to‐face interviews with all the participants. Interviews focused on physical and emotional experiences with the digoxin injection, experience with counseling related to the injection and fetal demise and understanding of digoxin's purpose. We recorded and transcribed the interviews. We also collected demographic data and abortion care characteristics data such as age, gestational age, history of prior abortion, indication for abortion, and doses of misoprostol administered to complete the abortion.

### 2.3 Data Analysis

Qualitative data were analyzed by an experienced qualitative research consultant with various past experiences in analyzing qualitative data. We continued to enroll participants and conduct interviews until we achieved thematic saturation, which the two coders agreed occurred with 20 interviews. Using a thematic analysis on One Code software, we analyzed transcripts, identified the themes from interviews, and stopped recruitment when we reached thematic saturation.

## 3 Results

A total of 20 women consented to participate and completed the qualitative interview. The mean maternal age was 22.8 years, and the median gestational age of the participants was 23 weeks (Table 1). Majority of the participants (63.2%) had a primary level of educational status, followed by another 21.1% (4/20) who had a secondary educational level. Maternal health and congenital anomaly were the commonest indication for abortion accounting for 40% (8/20) and 30% (6/20), respectively. For all participants, induced fetal demise was provided with intra‐amniotic digoxin.

**TABLE 1:** Sociodemographic and medication abortion care characteristics of Ethiopian women who had later medication abortion with induced fetal demise using Digoxin injection, 2023.

Variable	Category	*n*	%
Maternal age	Mean	22.8	
Gestational age weeks	Median	23	
Number of prior abortion	No prior abortion	18	90
	Had prior abortion	2	10
Educational level	Uneducated	1	5.3
	Primary	12	63.2
	Secondary	4	21.1
	College and above	2	10.5
Indication for abortion	Maternal health	8	40.0
	Congenital anomaly	6	30.0
	Rape	5	15.0
	Minor (age below 18)	1	5

When it comes to analysis of the qualitative data, the two overarching themes from the qualitative interviews were as follows: (1) emotional discomfort and varied understanding of digoxin's purpose; (2) digoxin injection‐related physical discomfort.

1. Emotional discomfort and varied understanding of digoxin's purpose

All women who went through the procedure had experienced feelings ranging from some level of stress to a high degree of physiological pressure. More than 50% of the participants knew the purpose of inducing fetal demise but a significant proportion of the participants, 8/20 (40%), described induced fetal demise as a procedure to shorten the abortion process. The psychological pain of having to carry a demised fetus for many hours till expulsion was a shared feeling by majority of the participants.

I didn't think it would happen like this. I thought my abortion would be completed with the abortifacient medication alone, like in my previous abortion.

Participant 3

It was difficult! Carrying a dead fetus for longer time before it finally went out was difficult.

Participant 5

That doesn't work. It didn't start the abortion process. The injection only cessation of the fetal heart beat.

Participant 1

1. Digoxin injection‐related physical discomfort

Majority of the respondents had cramps after the injection and few expressed sense of lower abdominal pushing down pain. However, a single participant (P4) experienced no pain, and another participant (P3) reported little pain. Few participants (P7&P16) experienced vomiting and dizziness following the administration of intra‐amniotic digoxin.

It hurts ‐abdominal pain and cramp‐but it is okay I understand the fetus will not survive.

Participant 8

## 4 Discussion

In the present study, discomfort of carrying a dead fetus after induced fetal demise until the abortion process was completed was a shared feeling by the participants. A significant proportion of them (8/20, 40%) described induced fetal demise as a procedure to shorten the abortion process. Majority of the respondents complained abdominal pain and cramps after the injection and few expressed sense of lower abdominal pushing down pain.

Digoxin is relatively safe feticide drug. However, the medical community remains divided about the routine use of digoxin in abortion care [12]. Patients’ experiences with digoxin are particularly relevant when considering whether it should be administered, yet data regarding patient preference for and experiences with digoxin are limited [13]. In this study, participants had a shared feeling of recognizing that they carry a dead fetus in their uterus. Majority of the respondents in our study complained abdominal pain and cramps after the injection and few expressed sense of lower abdominal pushing down pain. One participant (P4) experienced no pain, and another participant (P3) reported little pain. Few participants also experienced vomiting and dizziness following the administration of feticidal medications. In the most recent qualitative study in this topic, in which 20 participants were interviewed, it was found that most participants had significantly negative experiences with digoxin though many of the participants also reflected positive aspects of the injection intermingled with those negative experiences. The study further found that many women believed that digoxin would make their D&E procedure safer and easier [9].

The findings about participants’ low satisfaction with digoxin administration in our study are consistent with previous reports [9, 13, 14]. However, without comparative analysis of level of satisfaction with other feticide agents, it is too difficult to conclude as to whether digoxin is less or more preferred by abortion clients compared to the other procedures, for example, fetal intracardiac lidocaine injection. Future studies should explore this and come up with firm recommendation. A combined patients’ and providers’ preference using a mixed‐method study design would be an ideal approach to address this.

Despite uniform counseling about digoxin injection in the present study, subjects’ level of knowledge about the purpose of digoxin administration was not uniform (40% of the participants perceived that digoxin started the abortion process instead of inducing fetal demise). This finding is similar to the findings of the most recent qualitative study on patients’ experience with digoxin injection, which showed that many women believed that digoxin would make their D&E procedure safer and easier [9]. Although the abortion procedure in the previous qualitative study is different from that in our study (D&E vs. medication abortion), the findings of women's negative experience with digoxin injections complicated with a wrong belief that digoxin facilitates the abortion process are identical in both studies. This further demonstrates that maybe patients’ experiences with digoxin as a step before abortion care are not dependent on the method of abortion.

Strengths of our study included no delay between medication abortion procedure and time of interview, which was reported as a limitation in previous studies. In the present study, the participants were interviewed immediately after the abortion procedure and before they were discharged from the hospital. Among the main limitations of our study are lack of follow‐up information on the participants experience with digoxin and a single institution research/conducted in a single center. Including providers’ experience and attitude towards digoxin administration in our study could have further enabled us to generate more robust evidence to inform the medical community that remains divided about the routine use of digoxin in abortion care.

## 5 Conclusion

Findings of positive aspects of digoxin injection intermingled with those negative experiences (physical and emotional discomforts) in our study are similar to findings documented in prior studies. Providers need to make sure that adequate pre‐procedure counseling on digoxin injections has been provided, including ruling out misunderstanding about induced fetal demise. We recommend further comparative analytic study which should include providers experience as well as women's experience with other options of inducing fetal demise, for example, intracardiac lidocaine.

## Author Contributions

Abraham Fessehaye Sium and Tesfaye Tufa developed the concept and design of the project. Abraham Fessehaye Sium and Tesfaye Tufa contributed the data collection. Abraham Fessehaye Sium and Abrham Getachew contributed data analysis and subsequent interpretation of the analyzed data. Abraham Fessehaye Sium, Abrham Getachew, and Tesfaye Tufa contributed manuscript write‐up. All authors checked the manuscript for intellectual contents. The final manuscript is approved for submission by all authors.

## Disclosure

Abraham Fessehaye Sium is an Editorial Board member of Public Health Challenges and co‐author of this article. He was excluded from editorial decision‐making related to the acceptance of this article for publication in the journal.

## Ethics Statement

Ethical clearance was obtained from St. Paul's Hospital Millennium Medical College IRB. Written informed conscent was obtained from study participants. Participants did not receive any gift in‐exchange after completing their interviews. We coded interviews using thematic analysis as they were completed.

## Consent

Informed consent was not obtained from study participants. Written informed consent was obtained from each participant before conducting the interviews.

## Conflicts of Interest

The authors declare no conflicts of interest.

## Data Availability

All data generated or analyzed during this study are included in this published article.
